# Effects of computerized cognitive training on neuroimaging outcomes in older adults: a systematic review

**DOI:** 10.1186/s12877-017-0529-x

**Published:** 2017-07-10

**Authors:** Lisanne F. ten Brinke, Jennifer C. Davis, Cindy K. Barha, Teresa Liu-Ambrose

**Affiliations:** Aging, Mobility, and Cognitive Neuroscience Laboratory, Department of Physical Therapy, Djavad Mowafaghian Centre for Brain Health, University of British Columbia, 2215 Wesbrook Mall, Vancouver, BC V6T 1Z3 Canada

**Keywords:** Computerized cognitive training, Neuroimaging, Brain structure, Brain function, Older adults

## Abstract

**Background:**

Worldwide, the population is aging and the number of individuals diagnosed with dementia is rising rapidly. Currently, there are no effective pharmaceutical cures. Hence, identifying lifestyle approaches that may prevent, delay, or treat cognitive impairment and dementia in older adults is becoming increasingly important. Computerized Cognitive Training (CCT) is a promising strategy to combat cognitive decline. Yet, the underlying mechanisms of the effect of CCT on cognition remain poorly understood. Hence, the primary objective of this systematic review was to examine peer-reviewed literature ascertaining the effect of CCT on both structural and functional neuroimaging measures among older adults to gain insight into the underlying mechanisms by which CCT may benefit cognitive function.

**Methods:**

In accordance with PRISMA guidelines, we used the following databases: MEDLINE, EMBASE, and CINAHL. Two independent reviewers abstracted data using pre-defined terms. These included: main study characteristics such as the type of training (i.e., single- versus multi-domain), participant demographics (age ≥ 50 years; no psychiatric conditions), and the inclusion of neuroimaging outcomes. The Physiotherapy Evidence Database (PEDro) scale was used to assess quality of all studies included in this systematic review.

**Results:**

Nine studies were included in this systematic review, with four studies including multiple MRI sequences. Results of this systematic review are mixed: CCT was found to increase and decrease both brain structure and function in older adults. In addition, depending on region of interest, both increases and decreases in structure and function were associated with behavioural performance.

**Conclusions:**

Of all studies included in this systematic review, results from the highest quality studies, which were two randomized controlled trials, demonstrated that multi-domain CCT could lead to increases in hippocampal functional connectivity. Further high quality studies that include an active control, a sample size calculation, and an appropriate training dosage, are needed to confirm these findings and their relation to cognition.

## Background

With our ageing population, the incidence of dementia is rising rapidly. Currently, over 47 million people worldwide are diagnosed with dementia and this number is expected to triple by 2050 [1]. In 2010 it was estimated that the worldwide cost of dementia was 604 billion US dollars [1]. Thus it is imperative to find strategies that promote cognitive healthy aging to minimize the projected societal, health, and economic burden by reducing or delaying the potential progression to mild cognitive impairment or dementia.

Currently, there is no pharmaceutical cure for dementia. As such, identifying lifestyle approaches that may prevent, delay, or even treat cognitive impairment and dementia in older adults is becoming increasingly important [2]. Even when an effective pharmacological therapy is available, lifestyle approaches (i.e., exercise, nutrition, and cognitive training) can be used in conjunction as lifestyle interventions result in multidimensional benefits [3]. In recent years, there is growing interest in complex mental activity as a strategy to promote healthy cognitive aging. Complex mental activity comprises all activities that are cognitively challenging for an individual [4], such as memory and executive functioning training, or dance. A meta-analysis of human cohort studies provides robust evidence that complex patterns of mental activity in early, mid-life, and late-life stages is associated with a significant reduction in dementia incidence [5]. Furthermore, they found an association between increased levels of complex mental activity in late life and lower dementia rates, independent of other predictors. Finally, it showed a dose-response relationship between the amount of complex mental activities in late life and dementia risk [5].

Computerized cognitive training (CCT) is one example of complex mental activity that could be used to promote healthy cognitive aging. CCT is defined as cognitive training on an individual electronic device (e.g., computer, laptop, tablet/iPad) that requires a physical response such as a button press, and excludes training that primarily requires an individual to perform two tasks simultaneously, in order to compare performance with single-task conditions (i.e., dual-task training). Notably, CCT is an approach that could be used by those who are limited in their ability to physically participate in other strategies, such as exercise. A meta-analyses shows that CCT improved overall cognitive performance in older adults [6]. Specifically it showed improvements in verbal and non-verbal memory, working memory, processing speed, and visuospatial skills [6]. Recent randomized controlled trials (RCT’s) of CCT in older adults showed that both two and three months of training resulted in improved global cognition compared with an active control group [7, 8]. Additionally, an RCT showed that CCT resulted in improvements in memory and processing speed which were still visible twelve months post-training [7], and shows that CCT is able to maintain its benefits. Playing a real-time strategy video game for 23.5 h improved performance in executive functions, indicating transfer of training after participating in complex mental activities [9]. Thus, current evidence suggests that CCT is a promising strategy for promoting healthy cognitive aging.

Cognitive training is based on the notion that the brain, even with age, can change for the better, if given the appropriate environmental stimuli, thoughts, and emotions [10]. This capacity of the brain is called “neuroplasticity”. In the same way that physical training improves physical abilities, cognitive training (or brain training) may induce neuroplastic changes in the brain, resulting in improved cognitive abilities. One of the fundamental principles of neuroplasticity is the concept of synaptic plasticity – the notion that individual connections within the brain are constantly being removed or recreated, largely dependent upon how they are used [11]. Cognitive training aims to harness this principle of neuroplasticity by using guided practice on a set of tasks related to memory, attention, or other cognitive processes.

To gain more insight in what potential neuroplastic changes CCT may induce; incorporating different neuroimaging techniques in studies could be a good approach to help demonstrate these changes in the brain. For example, synaptic plasticity as a result of stimulation by CCT could potentially be captured by functional connectivity, measured with resting-state functional magnetic resonance imaging (rsfMRI), by strengthening connections within and between networks [12]. To date, it is not well established how CCT impacts regional brain volume, functional activity, and functional or structural connectivity in older adults. Although work has been done among younger adults illustrating changes in functional activity in the middle frontal gyrus and superior and inferior parietal cortices after working memory training [13], these findings don’t necessarily translate to an older adult population. Therefore, gaps remain in understanding the underlying mechanisms of training-induced neuroplasticity in older adults. Addressing this knowledge void, this systematic review aims to ascertain the mechanisms by which CCT exerts an impact on brain structure and function by using different neuroimaging techniques such as volumetric magnetic resonance imaging (MRI), task-based functional MRI (fMRI), rsfMRI, and diffusion tensor imaging (DTI). Through understanding the underlying neural mechanisms of CCT, our goal is to provide knowledge on how to design improved and targeted interventions that help combat or prevent cognitive decline throughout life.

## Methods

### Search strategy

**Fig. 1:**
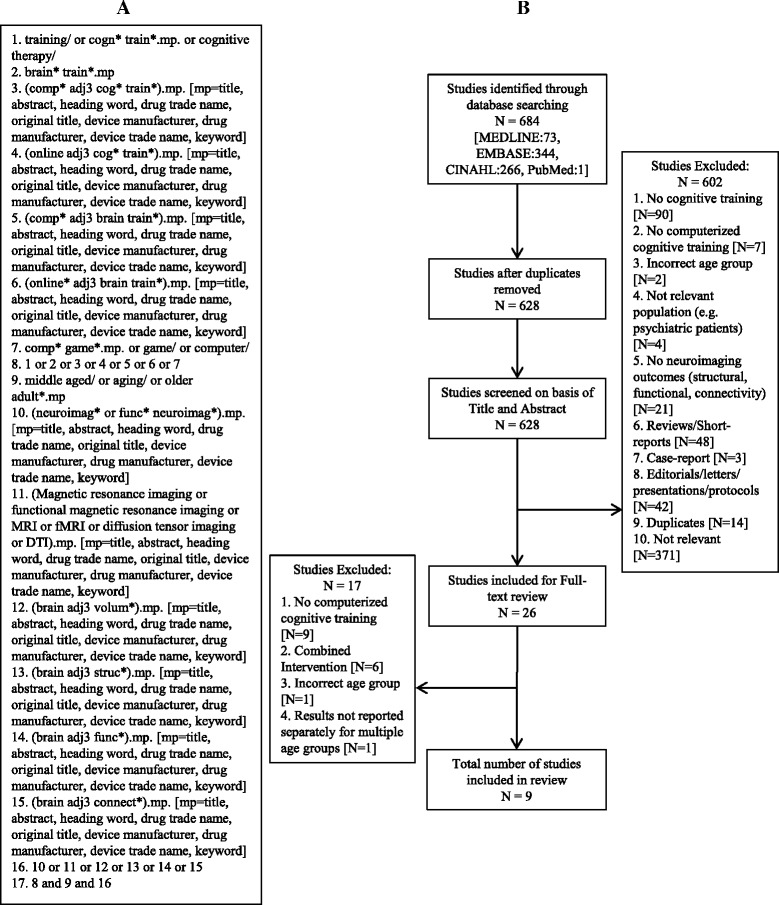
(**a**) Search Strategy retrieved from Ovid; (**b**) Exclusion pathway for study selection

In accordance with the Preferred Reporting Items for Systematic reviews and Meta-Analyses (PRISMA) statement [14], we conducted a comprehensive search of MEDLINE, EMBASE, and CINAHL databases to identify all the studies that investigated neuroimaging outcomes resulting from CCT interventions. We limited our search to adults aged 55 years and older with and without cognitive impairment, who have not been diagnosed with dementia. We did not limit the search based on publication date, as CCT is a relative novel research topic. The final search (see Fig. 1a for search strategy) was done on July 7 (2016) and included a check for recent publications in PubMed.

### Study selection

We selected studies that had a CCT intervention with neuroimaging outcomes (e.g. volumetric structural MRI, functional MRI, DTI) in an older adult population (age 55 years and older). Study designs included in this systematic review were RCT’s and quasi-experimental studies. Studies that used samples of younger and older adults but reported group results separately were included in this systematic review. We included studies that focused on both single- and multi-domain CCT programs. We considered single-domain CCT training as training that targeted a specific cognitive ability, such as working memory. In contrast, multi-domain CCT was considered training that consisted of a series of tasks that targeted multiple cognitive abilities (e.g., executive functions and memory). We excluded studies that did not focus on CCT or studies that used CCT in combination with other types of intervention (e.g., non-CCT, exercise), reviews and short reports. A full list of exclusion criteria and the exclusion pathway is displayed in Fig. 1b. Critical review of titles and abstracts resulted in 26 articles for full-text review.

### Data extraction and quality assessment

We developed a list of data extraction items. This list included reference, study sample, study design, MRI magnet, neuroimaging outcomes, cognitive function measured, training program/task, cognitive domain trained, description of training, training frequency and duration, total hours of training, supervised/home-based training, and control group. Two authors [LTB and CKB] independently extracted the data from the included studies. Discrepancies were discussed and solved by two authors [JCD and TLA].

The Physiotherapy Evidence Database (PEDro) scale [15] was used to assess the quality of the included studies. We [LTB and TLA] added three additional items to the PEDro scale to ensure a proper assessment of intervention studies using neuroimaging outcomes. These three items included were: 1) cognition measured to assist the interpretation of neuroimaging results; 2) sample size calculation; and 3) compliance reported (yes/no). To answer the items in the quality assessment, we used a ‘+’ for items that were present and a ‘-‘ for items that were absent. The quality assessment was performed independently by two authors [LTB and CKB]. Discrepancies were discussed and reviewed by two authors [JCD and TLA]. Consensus between two authors [LTB and CKB] was achieved after discussion (K=0.98). Because item one of the PEDro scale is related to external validity, it is not included in the overall PEDro score. Therefore, the maximum quality assessment score calculated by the PEDro was 10 points (each ‘+’ indicates one point), and will be reported in the results. Studies with a PEDro score of 6/10 or higher were considered studies of moderate to high quality. The additional item list had a maximum score of three points and trends from this list will be descriptively discussed in the results.

## Results

### Overview of studies included

Of the 684 articles identified, nine were included in this systematic review (Table 1). These nine papers included four RCT’s [16–19] and five quasi-experimental studies [20–24]; all nine studies had a different study duration. Details of the interventions included are provided in Table 2. The results are categorized into four categories: 1) Volumetric structural imaging (*n* = 4) [16, 19, 20, 22]; 2) Task-based fMRI (*n* = 3) [18, 21, 22]; 3) Connectivity (*n* = 7) [16, 17, 19, 20, 22–24]; and 4) Correlation between imaging outcomes and cognitive function outcomes (*n* = 8) [16–22, 24], (Table 3). Results are reported in order of study quality, starting with the highest quality.

**Table 1:** Characteristics of studies included

Reference	Study Sample^a^	Study Design Length of follow-up	MRI Magnet	Neuroimaging Outcome Measures	Cognition measured (test name)
Suo et al. [19] 2016	Older adults with MCI *N* = 100 70.1 ± 6.7 years Completed MRI: *N* = 79	RCT Assessments at baseline and 6 months	3 T	• Volumetric Structural MRI • Resting-state fMRI	Global Cognition (ADAS-Cog) [41] ○ Memory Domain ○ Executive Function ○ Attention-Speed
Rosen et al. [18] 2011	Older adults with MCI *N* = 12 74.34 ± 9.25 years Training *N* = 6 70.67 ± 10.58 years Control *N* = 6 78 ± 7.92 years	RCT Assessments conducted on average 72 ± 26 days apart	3 T	• Task-based fMRI • *Incidental Auditory-Verbal Repetition paradigm*	Memory (Repeatable Battery for the Assessment of Neuropsychological Status: RBANS) [42]
Lampit et al. [16] 2015	Healthy older adults: Subsample from Timecourse Trial *N* = 12 71.43 ± 7.48 years Training *N* = 7 72.3 ± 8 years Control *N* = 5 70.2 ± 6.7 years	RCT Assessments at baseline, 3 weeks: Follow Up 1(FU1), 3 months: Follow Up 2 (FU2) Secondary analysis	3 T	• Volumetric Structural MRI • Resting-state fMRI • Proton Magnetic Resonance Spectroscopy • DTI	Global Cognition: Composite of memory and information processing speed (Mindstreams battery) [43] as well as executive function (Average Mindstreams Stroop Interference test for Inhibition [43] and Cambridge Neuropsychological Test Automated Battery (CANTAB) Stockings of Cambridge problems solving) [44, 45]
Belleville et al. [21] 2014	Healthy community-dwelling older adults *N* = 40 69 ± 6.27 years Training group 1 *N* = 12 68.58 ± 8.16 years Training group 2 *N* = 14 69.57 ± 5.81 years Training group 3 *N* = 14 68.79 ± 5.13 years	Quasi-experimental Pre-post Assessments 1 week before and 1 week after training	3 T	• Task-based fMRI • *Alphanumeric equation task* • *Visual detection task* Tasks performed as single-task and dual-task	Reaction Time (Alphanumeric equation task and visual detection task) Accuracy (Alphanumeric equation task and visual detection task)
Lin et al. [17] 2014	Older adults with a history of a stroke *N* = 34 69.21 ± 4.93 years Training *N* = 16 62.4 ± 6.0 years Control *N* = 18 63.2 ± 5.7 years	RCT Assessments at baseline and 10 weeks	3 T	• Resting state fMRI	Memory (Wechsler Memory Scale) [46] Executive Function (Trail Making Test) [47]
Strenziok et al. [24] 2014	Healthy older adults *N* = 42 69.21 ± 4.93 years Training group 1 *N* = 14 69.70 ± 6.9 years Training group 2 *N* = 14 68.52 ± 5.6 years Training group 3 *N* = 14 69.41 ± 2.3 years	Quasi-experimental Pre-post Length of follow up: Not stated	Not stated	• Resting-state fMRI • DTI	Reasoning/Problem Solving (WAIS III Matrix Reasoning subtest, [48] Everyday problems Test, [49] Word Series and Letter Series Tests) [50] Episodic Memory (Wechsler Memory Scale Logical Memory Subtest) [51, 52] Spatial Working Memory (Information-processing Visuo-Spatial Delayed Match-to-Sample Test) [53, 54] Auditory Working Memory (Letter Number Sequencing subtest of WAIS III) [48]
Lövden et al. [23] 2010	Healthy older adults^b^: Subsample COGITO study *N* = 25 69.32 ± 3.12 years Training *N* = 12 68.9 ± 2.7 years Control *N* = 13 69.7 ± 3.5 years	Quasi-experimental Pre-post Training: Pre-post MRI on average 179 ± 25.2 days apart Control: Pre-post MRI on average 184 ± 15.0 days apart	1.5 T	• DTI	Spatial Working Memory (3-Back)^c^ Numerical Working Memory (Memory Updating)^c^ Figural-Spatial Episodic Memory (Object-Position Memory)^c^ Numerical Episodic Memory (Number-noun pairs)^c^ Verbal Episodic Memory (Wordlist)^c^ Perceptual Speed (Choice Reaction Task, Comparison tasks)^c^
Antonenko et al. [20] 2016	Healthy older adults *N* = 25 69 ± 6 years	Quasi-experimental Pre-post Assessments 1 day before (pre), 1 day after (post) and 1 month after (follow-up) training^d^	3 T	• Volumetric Structural MRI • DTI	Cued recall (3-alternative-forced-choice recall task (AFC); main outcome) [55] and recognition Episodic Memory control task (German Rey Auditory Verbal Learning Test) [56]
Heinzel et al. [22] 2014	Healthy older adults^c^ *N* = 19 65.95 ± 3.73 years	Quasi-experimental Pre-post Subset of 15 older individuals performed pre-post MRI Length of follow up: Not stated	3 T	• Volumetric Structural MRI • Task-based fMRI • *N*-back [57]: two runs (16 blocks/run) with 4 working memory loads (0, 1, 2, 3) • Functional Connectivity (PPI)	Relative Working Memory Training gain (*n*-Back) [57] Short-term memory (Digit span Fwd and Bwd WAIS III) [51] Processing Speed (Digit Symbol WAIS III, [51] D2 Test [58]) Executive Functions: Verbal Fluency (Controlled Oral Word Association Test) [59] Inhibition (Stroop) [60] Abstract Reasoning (Raven’s SPM [61], Figural Relations subtest [62])

*MRI* Magnetic Resonance Imaging, *DTI* Diffusion Tensor Imaging, *fMRI* functional Magnetic Resonance Imaging, *RCT* Randomized Controlled Trial

^a^Mean age ± standard deviation

^b^A sample of young adults was included in the study as well

^c^Behavioural outcomes only measured for intervention groups

^d^Only cognitive assessments at one month follow-up (no MRI)

**Table 2:** Details of the computerized cognitive training intervention for the studies included

Reference	Training program/ task Cognitive Domain Trained	Description of Training	Training Frequency Training Duration	Total hours of training Supervised/ Home-based	Control Group
Suo et al. [19] 2016	COGPACK Multidomain: memory, attention, response speed, executive functions, language	COGPACK: Exercises focused on memory, attention, response speed, executive functions, and language	26 weeks 52 sessions 90 min/session	78 Supervised	Active: watched videos on computer, followed by questions
Rosen et al. [18] *2011*	Posit Science Multidomain: processing speed, accuracy in auditory processing	Auditory verbal repetition paradigm: 7 exercises aimed at improving processing speed and accuracy in auditory processing	5 weeks 24 sessions 100 min/session	36 Home	Active: 3 different computer-based activities (listening to audiobooks, reading online news, playing visuospatial computer game)
Lampit et al. [16] 2015	COGPACK Multidomain: memory, attention, response speed, executive functions, language	Exercises focused on memory, attention, response speed, executive functions, and language	12 weeks 3×/week 60 min/session	36 Supervised	Active: viewed 7 National Geographic videos per session on a computer with multiple choice questions
Belleville et al. [21] 2014	Customized program Executive Function: Attention	*Alphanumeric equation task:* judge accuracy of visually presented letter and number equations. *Visual detection task:* detect the red rectangles (press a button) in a series of white and red rectangles Groups: 1. Single repeated: Complete both tasks individually (focused attention) 2. Divided fixed: Complete 2 tasks simultaneously with divided attention (50%) 3. Divided variable: Complete two tasks simultaneously with different attention allocation levels (80%, 50%, 20%)	2 weeks 3×/week 1 h/session	6 Supervised	No Control
Lin et al. [17] 2014	RehaCom Executive Function and memory	Computer-assisted exercise focused on memory and executive function	10 weeks 6×/week 60 min/session	60 Supervised	Passive
Strenziok et al. [24] 2014	Multidomain: Brain Fitness (BF): auditory perception; Space Fortress (SF): visuomotor and working memory Rise of Nation (RoN): attention, motor processing, working memory, reasoning, visuospatial short-term memory, task-switching	1. Brain Fitness (BF): Adaptive auditory perception computer game 2. Space Fortress (SF): Complex skill acquisition computer game 3. Rise of Nations (RoN): Off-the shelf real-time strategy computer game	6 weeks 36 sessions 60 min/session	36 Supervised + Home (50–50%)	No Control
Lövden et al. [23] 2010	Customized program Multidomain: working memory, episodic memory, perceptual speed	Working Memory (3-Back, Memory updating, Alpha span) Episodic memory (Object-position memory, Number-noun pairs, Word lists) Perceptual speed (Choice reaction tasks, Comparison Tasks)	>4 months Average of 100 ± 3.7 sessions 60 min/session	Average of 100 Supervised	Passive: Pre-post MRI only
Antonenko et al. [20] 2016	Object-location Learning Paradigm Memory	*Object-location Learning Paradigm:* Learn the correct spatial locations of buildings on a street map. Five blocks of 120 stimulus-location pairing with a response interval of 3 s. Each block was followed by a cued recall and a recognition task	3 consecutive days 5 learning blocks/day	Unknown Unspecified	No Control
Heinzel et al. [22] 2014	*n* **-**Back training Executive Function: Working Memory	Adaptive *n*-back training, 3 runs (12 blocks/run) each session. Difficulty level increased according to individual performance (higher working memory load, shortened interstimulus interval (ISI). ISI ranged from 1500 to 500 ms in steps of 500 ms.	4 weeks 3×/week 45 min/session	9 Supervised	No control

**Table 3:** Results for Imaging Outcome measures

Reference	Structural changes	Functional changes	Changes in connectivity	Cognition Outcome	Cognition related to imaging outcome
Suo et al. [19] 2016	Combined cognitive training and progressive resistance training led to increased cortical thickness in posterior cingulate cortex. Cognitive training alone led to atrophy.	-	Cognitive training groups showed Group X Time interaction indicating decreased connectivity between the posterior cingulate and superior frontal lobe (F(67) = 31.7, *p* < 0.001) and between the posterior cingulate and the anterior cingulate cortex (F(67) = 13.9, *p* < 0.001)^a^. Cognitive training group (alone or combined with exercise) showed a Group X Time interaction indicating increased connectivity between hippocampus and the left superior frontal lobe compared with non-computerized cognitive training (*p* = 0.012)^a^	Computerized cognitive training (alone and with resistance training): Memory domain: Group X Time interaction (F(90) = 5.7, *p* < 0.02) showing no decline in cognitive training group compared to non-cognitive training groups^a^ ADAS-Cog: No effect of cognitive training	Change in posterior cingulate grey matter correlated with improvement in the ADAS-Cog (*r* = 0.25, *p* = 0.030)^a^. Increased connectivity between hippocampus and superior frontal lobe was correlated with improved memory domain performance (*r* = 0.33, *p* = 0.005)^a^
Rosen et al. [18] 2011	-	Significant increase of activation in left anterior hippocampus in experimental group compared with controls.	-	A non-significant but greater gain in memory performance in experimental group compared with control group (*F*(1,10) = 4.76, *p* = 0.054). Change scores showed improved memory performance in intervention group compared with decrease in performance in the control group (t(10) = 2.61, *p* < .0027, Cohen’s *d* = 1.38)	Non-significant trend showing changes in hippocampal activation correlated positively with changes in memory score on RBANS in all participants (*r* = 0.49, *p* = 0.10, Cohen’s *d* = 1.14)
Lampit et al. [16] 2015	Significant increase in grey matter density in right post-central gyrus in training group compared with a decrease in control. Vertex-based analysis showed significant difference in rate of thickness change over time between training and control in both the left fusiform gyrus (*T* > 3.39) and the supramarginal and post-central gyri (*T* > 2.24).	-	Group x Time interaction showed functional connectivity decrease between posterior cingulate and right superior frontal gyrus in training group while functional connectivity increased in the control group (*p* = .006) at FU1. Group x Time interaction showed functional connectivity increase between right hippocampus and left superior temporal gyrus in CCT, while decreased in control at first FU1 (*p* = .029). No significant Group x Time interactions found for Magnetic Resonance Spectroscopy (MRS) and whole brain Diffusion Tensor Imaging (DTI)	Repeated-measured ANOVA showed improved global cognition in training group compared to control (Group X Time, *F* = 7.833, *p* = 0.003). Effect size on Global Cognition (*d* = 0.94 baseline versus FU1 and *d* = 2.18 baseline versus FU2)	Significant positive correlation between change in grey matter density in right post-central gyrus at FU2 and change in global cognition at FU1(*r* = 0.647, *p* = .023) and FU2 (*r* = 0.584*, p* = 0.046) in both training and control. Inverse correlation between functional connectivity between posterior cingulate and right superior frontal gyrus at FU1 and change in global cognition at FU2 (*r* = −.771, *p* = .003). Significant positive correlation in functional connectivity between the right hippocampus and left superior temporal gyrus at FU1 and change in global cognition at FU2 (*r* = 0.591, *p* = .043).
Belleville et al. [21] 2014	-	Single Repeated: *Alphanumeric single* *task:* Decreased post- training activation in inferior and right middle frontal gyrus (*t* = 5.91), left middle frontal gyrus (*t* = 4.57) and left thalamus (*t* = 5.37). *Visual detection single* *task*: no change *Dual task*: no change Divided Fixed *Alphanumeric single task:* no change *Visual detection single task:* Decreased post-training activation in right cerebellum (*t =* 4.73) and right middle occipital gyrus (*t* = 4.68) when performing the visual detection task. *Dual task (50/50): S*mall increase in post-training activation in right and left middle frontal gyrus (areas 11, 47; *t* = 4.41 and *t* = 4.52 respectively). Divided Variable *Alphanumeric single task:* no change *Visual detection single task:* no change *Dual task:* Significant increased activation in right middle frontal gyrus (area 10; for 20% attention allocation *t* = 5.35 and 50% attention allocation *t* = 4.78). No reduced post-training activation in 80% attention allocation.	-	*Alphanumeric single task:* All groups showed improved reaction time (RT; F(1,34) = 9.75, *p* < .001, η^2^ = .22) and accuracy (AC; F(1,34) = 14.8, *p* = .001, η^2^ = .30) *Visual detection single task*: No change *Dual task (cost score)* ^b^: Single repeated: No improvements in dual tasking Divided Fixed: Reduced dual-task cost (F(1,34) = 6.97, *p* < .001, η^2^ = .45) Divided Variable: Reduced dual-task cost and were able to modify attentional priority (F(2,33) = 5.17, *p* < .001, η^2^ = .34)	Single Repeated: *Alphanumeric single task:* Significant positive correlation between right inferior and middle frontal gyrus activation and reaction time (*r* = .56, *p* < .05). Divided Variable: Significant negative correlation (post training) between activation of right superior and middle frontal gyrus (Brodmann area 10) and attentional cost (*r* = −.55, *p* < .05)
Lin et al. [17] 2014	-	-	Training group: Significant increased functional connectivity in (all *p’*s < 0.005): 1.Left hippocampus-right inferior frontal gyrus 2.Left hippocampus-right middle frontal gyrus 3.Right hippocampus-left middle frontal gyrus 4.Right hippocampus-left inferior frontal gyrus 5.Right hippocampus-left superior frontal gyrus 6.Right hippocampus-left parietal lobe Control group: Significantly decreased functional connectivity (all *p*’s < 0.005): 1. Left hippocampus-right middle occipital gyrus 2. Right hippocampus-right posterior lobe or cerebellum 3. Right hippocampus-left superior temporal gyrus	Training group: 1. Significant improved scores on 5/7 subtests from Wechsler Memory Scale, namely: Mental control (*p* = 0.003), Logical memory (*p* < 0.001), Digits forward and backward (*p* = 0.014), Visual reproduction (*p* = 0.008), and Associated learning (*p* < 0.001). 2. Improved Memory quotient (*p* = 0.005) 3. Improved performance on Trail Making Test-A (*p* < 0.001) Control group: no significant changes between baseline and 10-week scores	Training group: significant positive correlations between (all *p*’s < 0.001): 1. Memory quotient and functional connectivity of left hippocampus-right frontal lobe (*r* = 0.64) 2. Memory quotient and functional connectivity of right hippocampus-left frontal lobe (*r* = 0.85) 3. Memory quotient and functional connectivity of right hippocampus-left parietal lobe (*r* = 0.79) 4. Trail Making Test-A score and functional connectivity of left hippocampus-right frontal lobe (*r* = 0.94) 5. Trail Making Test-A and functional connectivity of right hippocampus-left frontal lobe (*r* = 0.68) Control group: no significant correlations between cognition and functional connectivity^c^
Strenziok et al. [24] 2014	-	-	Ventral Network: Axial diffusivity (AD) in the right occipito-temporal white matter significantly increased after BF compared with a decrease after SF and RON (*p* < 0.05) Dorsal Network: Functional connectivity between right superior parietal cortex (SPC) and left posterior inferior temporal lobe (ITL) decreased in SF and increased in RON (*p* = 0.02). Functional connectivity between right SPC and left anterior ITL decreased in BF and showed an increase in RON (*p* = 0.03)	Univariate ANOVA showed main effects of training group: Reasoning on Everyday Problems Test: Main effect of training group (F(2,39) = 5.34, *p* < 0.01, partial η^2^ = 0.215). BF and SF showed improved performance after training and RON showed no effect. Spatial Working Memory: Main effect of training group (F(2,39) = 5.03, *p* < 0.001, partial η^2^ = 0.205). SF improved performance after training, RON decreased performance, and BF showed no effect. Matrix Reasoning: Main effect of training group (F(2,39) = 3.40, *p* < 0.044, partial η^2^ = 0.148). Largest gains seen in BF and a smaller gain in RON. The SF group showed a decrease in reasoning after training	Cognition and White Matter Integrity Positive correlation between change in thalamic AD and change in working memory performance in all participants (*r* = 0.44, *p* < 0.005). Negative correlation between changes in occipito-temporal AD and everyday problem solving (*r* = −0.32, *p* < .05) and spatial working memory accuracy (*r* = −0.35, *p* < .05). Negative correlation between changes in occipito-temporal-parietal AD and spatial working memory accuracy (*r* = −0.40, *p* < 0.05). Cognition & Functional Connectivity Positive correlation between changes in SPC-posterior ITL connectivity and changes in everyday problem solving time (*r* = −0.57, *p* < .001).
Lövden et al. [23] 2010	-	-	Mean Diffusivity (MD) Group X Time interaction found for segment 1 (genu) of corpus callosum, showing a decrease in MD (*t*(11) = 2.39, *p* = .036). No changes in control group Fractional Anisotropy (FA) Group X Time interaction found for segment 1 of corpus callosum, showing an increase in FA (*t*(11) = 3.12, *p* = .010)	Unknown: analysis combined younger and older subsets	Unknown: analysis combined younger and older subsets
Antonenko et al. [20] 2016	Hippocampal volume: no difference pre to post training (*p* = 0.505)		Mean Diffusivity (MD): A significant decrease in fornix MD was found at post-training compared with pre-training (*p* = 0.036). No difference in hippocampal MD from pre- to post-training (*p* = 0.669). Fractional Anisotropy (FA): A non-significant increase in fornix FA was found between pre- and post-training (*p* = 0.114)	*% Correct during training:* Task performance significantly improved in a curvilinear convex manner over the 3 training days learning	- Higher increase in fornix FA from pre to post assessment was significantly related to better average recall performance on the object-location task during training, at 1-day post and follow-up (*r* = 0.431, *p* = 0.031) - Change in fornix FA did not correlate with episodic memory performance on the control task (Rey Auditory Verbal Learning Test; *p* = 0.214) - Change in fornix MD did not correlate with recall performance *p* = 0.728 - Change in hippocampal MD or volume did not correlate with recall performance (*p* = 0.688 and *p* = 0.758, respectively)
Heinzel et al. [22] 2014	No significant change in grey matter volume of working memory network post training (*t*(14)= 0.83, *p* = .421)	No significant 2(time) ×3(working memory load) interaction (*F* = .24, *p* = .714, partial η^2^ = .024). Significant main effect of time (*F* = 12.68, *p* = .003, partial η^2^ = .475) driven by BOLD decrease in 1-back (*t* = .99, *p* = .029).	A 2(time)×3(load) repeated measures ANOVA showed no changes in connectivity in working memory network (F(2,28) = 1.08, *p* = .355, partial η^2^ = .071)	*n*-Back: paired t-tests showed improved performance on 1-Back (t(18) = 3.37, *p* = .003), 2-ack (t(18) = 7.47, *p* < .001), and 3-Back (t(18) = 4.86, *p* < .001)^d^. Repeated-measures MANOVA (factor time) showed improvements in neuropsychological measures after training. Post hoc paired t-tests showed improvements in Digit Span Fwd (t(18) = 2.97, *p* = 0.008), D2 test (t(18) = 6.48, *p* < 0.001), Digit Symbol (t(18) = 2.76, *p* = 0.013), Stroop Interference (t(18) = 3.28, *p* = 0.004), and Figural Relations (t(18) = 4.73, *p* < 0.001). No improvements after training were found in Digit Span Bwd, Verbal Fluency, and Raven’s SPM.^d^	Non-significant trend between BOLD activation at baseline and relative improvement in Digit Span Fwd (*r* = .43, *p* = .067)

^a^This study was a full factorial design

^b^This dual-task cost represents the proportional loss of performance in the dual-task condition as a function of performance in the single-task condition. A larger score represents a larger dual-task cost

^c^Not specified whether correlations were based on change scores or scores at week 10

^d^Results reported for all older participants (*N* = 19)

### Structural imaging (*n* = 4)

Four studies [16, 19, 20, 22] reported volumetric and cortical thickness outcomes (Table 3). A randomized controlled study (full factorial design) multi-domain cognitive training study using Cogpack [19], older adults with mild cognitive impairment (MCI) trained for a total of 78 h over a period of 6 months under supervision. Combined cognitive training with resistance training resulted in increased cortical thickness in the posterior cingulate cortex. However, in the same study they found that cognitive training alone led to a decrease in the posterior cingulate cortex thickness. However, there was no difference in decrease in thickness compared with the control group.

In addition, a twelve-week supervised multi-domain CCT study [16] using the same program (CogPack) showed that 36 h of training resulted in an increase in grey matter density in the right post-central gyrus compared with a decrease in the active control group. Additionally, the training resulted in a difference in rate of thickness change over time in both the left fusiform gyrus and the supramarginal and post-central gyri.

In contrast, in an object-location learning paradigm study [20] participants performed training on three consecutive days where they had to learn the correct spatial location of buildings on a street map. On each training day, the training was followed by a cued recall and recognition task. Hippocampal volumes was measured pre- and post-training. The authors found that the object-location learning paradigm did not lead to changes in hippocampal volume.

In another quasi-experimental study [22], participants performed an adaptive working memory training (*n*-Back) for twelve 45-min sessions over 4 weeks. Difficulty level of the training was based on individual performance and increases over time. Results showed that the training did not result in changes in grey matter volume in the working memory network.

In summary, one RCT [19] found cortical thinning as a result of cognitive training alone. In contrast, another RCT [16] found an increase in grey matter density following training. Finally, one study [22] found that cognitive training did not result in changes in grey matter, and one study [20] found that cognitive training did not lead to changes in hippocampal volume.

### Task-based fMRI (*n* = 3)

Three [18, 21, 22] of the eight included studies examined the effect of a CCT intervention on brain function as measured via task-based fMRI (Table 3). An RCT [18] showed that 2200 min of cognitive training over a period of 5 weeks resulted in a significant increase in left anterior hippocampus activity compared with an active control group. The cognitive training consisted of seven games aimed to improve auditory processing speed and accuracy. Task difficulty was adjusted throughout the training based on individual performance. The active control group performed computer-based activities such as reading online newspapers and playing computer games targeting visuospatial abilities.

A two-week quasi-experimental study looked at focused and divided (fixed and variable) attention training [21]. In the focused attention training, two tasks (i.e., alphanumeric task and a visual detection task) were performed back to back but separate so participants focused on one task at a time. In the divided attention training, participants performed two tasks at the same time with an equal amount of attention (fixed) or under different attention allocations (variable). Results showed that training a single alphanumeric task for 6 h over two weeks decreased activation in the inferior and right middle frontal gyrus, in the left middle frontal gyrus and in the left thalamus. No differences in functional brain activation were found after performing the single visual detection task or the in the dual task condition. Participants who were assigned to training where they performed both the alphanumeric task and the visual detection task at the same time (i.e., dual task) did not show differences in performance during the alphanumeric task in the scanner. However, participants showed decreased functional brain activation at post-training compared with pre-training in the cerebellum and right middle occipital gyrus during the single visual detection task. Additionally, participants showed a slight increase in activation in both the right and left middle frontal gyrus. Finally, participants who were assigned to the training group where they had to perform dual tasks under different attention allocation levels (i.e., 80%, 50%, or 20%), showed increased activation in the right middle frontal gyrus (area 10) for 20% and 50% attention allocation when performing the dual task. No significant changes in functional brain activation were found during the 80% attention allocation task, neither during the alphanumeric single task, nor during the visual detection single task performance.

In an adaptive *n*-back training program [22], participants performed 12 sessions of approximately 45 min each over 4 weeks. The difficulty level of the training was based on individual performance and was increased across training sessions by increasing working memory load and decreasing the interstimulus interval. Results of this study showed a non-significant time (2) by working memory load (3) interaction, with a significant main effect of time. This main effect of time demonstrates a reduction in working memory network functional brain activity measured by the Blood Oxygen Level Dependent (BOLD) signal after 12 training sessions. Only decreases in the 1-back (and not 2-back or 3-back) condition were significant, which indicates this main effect of time is driven by the BOLD signal during the 1-back condition.

In summary, an RCT [18] showed that 2200 min of CCT resulted in increased in left anterior hippocampus activity compared with an active control group. One quasi-experimental study [21] showed that depending on the task and region of interest, all training conditions resulted in both increased and decreased activity. Finally, a second quasi-experimental study [22] found that 12 sessions of *n*-back training resulted in a significant decrease in working memory activity; however decrease in activity was driven by performance on the 1-back condition.

### Connectivity

#### Resting-state fMRI (*n* = 5)

Five studies [16, 17, 19, 22, 24] looked at changes in functional connectivity after CCT (Table 3). An RCT [19] examined the effect of progressive resistance training (PRT), computerized multi-domain cognitive training (CCT), or a combined intervention on brain structure and function in older adults with mild cognitive impairment (MCI). The study duration was 26 weeks, with a total of 78 h of training. In the cognitive training groups (i.e., PRT + CCT, and CCT + Sham), the posterior cingulate cortex showed significant decreases in resting-state functional connectivity with both the superior frontal lobe and the anterior cingulate cortex. In addition, increases in resting-state functional connectivity between the hippocampus and the left superior frontal lobe were found compared with groups without CCT.

A second RCT of 12 weeks of multimodal CCT [16] showed that 36 h of cognitive training resulted in decreases in resting-state functional connectivity between the posterior cingulate and the right superior frontal gyrus, while the control group showed significant increases in resting-state functional connectivity. In contrast, CCT resulted in increased resting-state functional connectivity between the right hippocampus and the left superior temporal gyrus compared with a decrease in connectivity in the control group.

Another RCT [17] looked at the effects of a 10-week computer assisted training focused on executive functioning and memory in older adults with a history of stroke. The authors found that training, compared with a passive control group, significantly increased resting-state functional connectivity in multiple areas. The left hippocampus showed significantly increased connectivity with the right inferior frontal gyrus and the right middle frontal gyrus. Additionally, the right hippocampus showed increased resting-state functional connectivity with the left middle frontal gyrus, the left inferior frontal gyrus, the left superior frontal gyrus and the left parietal lobe. In contrast, the control group showed significant decreases in resting-state functional connectivity over the 10 weeks (see Table 3 for connectivity decreases).

A quasi experiment investigating the effect of three different computer programs [24] found an increased resting-state functional connectivity in the dorsal network between the right superior parietal cortex (SPC) and left posterior inferior temporal lobe (ITL) in Rise Of Nation (RON) compared with a decrease in Space Fortress (SF). Finally, Brain Fitness (BF) resulted in significantly decreased resting-state functional connectivity between the right SPC and the left anterior ITL compared with an increase in RON.

Finally, a quasi-experimental study [22] looking at the effects of an adaptive *n*-back training program in older adults found that the 5-week training did not result in changes in task-based functional connectivity in the working memory network.

#### Structural connectivity (*n* = 4)

Four studies [16, 20, 23, 24] examined changes in structural connectivity, using DTI, after CCT (Table 3). Whole brain diffusion tensor imaging (DTI) of an RCT of 12 weeks of multimodal CCT [16] showed that 36 h of cognitive training did not result in changes in structural connectivity after training.

A quasi-experiment in healthy older adults looked at the effect of three different training protocols on brain structure [24]. The participants trained for 36 h over a period of 6 weeks; half of the training was supervised, and the other half was performed at their own homes. One training group performed BF, an auditory perception game; the second training group performed SF, a complex skill acquisition game focused on visuomotor and working memory skills; and the third group performed RON, an off-the shelf real-time strategy game focused on for example attention, motor processing, working memory and reasoning. The authors found changes in the ventral and dorsal network. Axial diffusivity (AD) was increased in the right occipito-temporal white matter in the BF group, compared with a decrease in SF and RON.

Another quasi-experimental study [23] of approximately 100 h of multi-domain cognitive training in both young and healthy older adults performed Diffusion Tensor Imaging (DTI) to look at the effects of training on structural connectivity in the brain. Result showed a significant decrease in MD in the genu of the corpus callosum compared with a passive control group who showed no changes in MD. They also found a significant increase of fractional anisotropy (FA) in the genu of the corpus callosum compared with the control group.

Diffusion Tensor Imaging results from a third quasi-experimental study [20] that involved 3 consecutive days of training an object-location learning paradigm, showed that the 3-day training resulted in a significant decrease in mean diffusivity (MD) in the fornix at post-training compared with pre-training. No changes in MD were found in the hippocampus as a result of the training. In addition, the results showed an increase in FA in the fornix, however this increase was not significant.

In summary, the seven [16, 17, 19, 20, 22–24] above mentioned rsfMRI and DTI studies showed both increases and decreases in functional and structural connectivity after CCT. The variety in study protocol (i.e., training type, duration) and the regions of interest chosen for neuroimaging analysis makes the comparison between studies difficult.

### Correlation between imaging outcomes and cognitive function outcomes (*n* = 8)

Eight studies [16–22, 24] assessed the association between cognitive performance and neuroimaging findings (Table 3). An RCT in older adults with a history of stroke [19] found that increases in posterior cingulate grey matter were associated with improvements in global cognition. Additionally, a cognitive training by time interaction showed that the increased connectivity between the hippocampus and the left superior frontal lobe was related to increased memory domain performance. However, this interaction takes into account all training groups that had a cognitive training component (i.e., also cognitive training combined with resistance training). The inclusion of the combination group might have influenced this interaction.

In contrast, an RCT looking at CCT in older adults with MCI [18] found no significant correlations between neuroimaging and cognitive results. However, the authors found a non-significant trend suggesting that, in both groups, increases in hippocampal activity might be related to improved memory scores on the RBANS.

An RCT of multimodal CCT [16] found that increased grey matter density in the right posterior central gyrus was associated with improved global cognition at 3 weeks and 3 months. This association was found in both the training and control group. In addition, it was found that a decrease in resting-state functional connectivity between the posterior cingulate and the superior frontal gyrus after 3 weeks of training was related to an increased change in global cognition after 3 months of training. Increased resting-state functional connectivity between the right hippocampus and the left superior temporal gyrus measures after three weeks of training was associated with increases in global cognition after 3 months of training.

A quasi-experimental study [21] found that in participants performing the alphanumeric task in the single task condition (i.e., focus on one task at the time), there was a significant positive correlation between both the right inferior and the middle frontal gyrus activation and reaction time. Thus shorter reaction time (i.e., better performance) was associated with a decrease in brain activation. In the divided variable condition (i.e., dual task with different attention allocation levels), there was a negative correlation between activation of the right superior and middle frontal gyrus and attentional cost post training. This correlation indicates that a better training performance (i.e., lower attentional cost during dual task performance) was associated with higher levels of brain activation.

An RCT in older adults with a history of stroke [17] revealed that in the multimodal cognitive training group, resting-state functional connectivity between the left hippocampus and both the right frontal lobe and right frontal lobe, was associated with improved performance in memory executive function respectively. Additionally, increases in resting-state functional connectivity between the right hippocampus and the left frontal lobe and the left parietal lobe were associated with increases of memory and executive functioning. No significant associations between functional connectivity and behavioural performance were found in the control group.

A quasi-experimental study looking at the effect of three different types of cognitive training on brain structure and function [24] found that in the BF training group an increase in thalamic AD was associated with an increase in working memory performance. By comparing BF and SF, the authors found that an increase in occipito-temporal AD was associated with a decrease in everyday problem solving time. Additionally, they found an association between the increase in both the occipito-temporal AD and occipito-temporal-parietal AD and accuracy of spatial working memory tasks, indicating that a greater AD was associated with a smaller increase in accuracy on the memory task. Finally, looking at the contrast between SF and RON, functional connectivity decreases between the superior parietal cortex (SPC) and the posterior inferior temporal lobe (ITL) were related to better performance on every day problem solving tasks (i.e., decrease in time for task completion).

In another quasi-experimental study [20], participants training for 3 consecutive days on an object-location learning paradigm. The authors found that the previous mentioned increase in fornix FA on the post-test compared with pre-test was significantly associated with better recall performance. Thus, a higher increase in fornix FA over the course of the training resulted in a better recall performance on the object-location learning paradigm task. Changes in fornix MD, hippocampal MD, and hippocampal volume were not associated with recall performance. Performance on the episodic memory control task was not associated with changes in fornix FA.

The last quasi-experimental study [22] looked at changes in short term memory (digit span) and found a non-significant trend between task-based functional activation at baseline and improvement in digit span, which indicates that an increased activation might lead to increased short term memory performance.

In summary, eight [16–22, 24] of the nine studies [16–24] included demonstrated an association between changes in neuroimaging measures (volumetric or connectivity) and changes in behavioural outcomes. Depending on the region of interest (i.e., both volumetric and connectivity), both increases and decreases in activity resulted in improved cognitive performance. One study [18] found no significant association between neuroimaging and behavioural measures. One study [23] did not report the association between neuroimaging and cognition in older adults specifically.

### Quality assessment of the included studies

The quality of studies included in this systematic review varied substantially (Table 4). On average, the nine included studies met 7 of the 11 PEDro criteria. Two studies of the highest quality [18, 19] meeting 9 of the 10 PEDro criteria; however, five [17, 20, 22–24] studies failed to meet five or more study quality criteria. Item 11 (i.e., included point measures and variability measures) was met for all nine studies. Item 8 (key outcome measured for 85% of subjects) and nine (outcome data analyzed by intention to treat) were met by seven of the nine studies [18–24]. Item 6, (i.e., blinding of all who administered the training) commonly received a negative response (i.e., one of the studies [19] blinded training administers). Frequent issues were failure to meet or report: 1) allocation concealment (*n* = 4) [20, 22–24]; 2) blinding of all subjects (*n* = 6) [17, 20–24]; 3) blinding of all who administered the training (*n* = 8) [16–18, 20–24]; 4) blinding of assessors who measured at least one key outcome (*n* = 5) [20–24]; and 5) between-group statistical comparisons for at least one key outcome (*n* = 4) [17, 20–22]. Item 9 (participants with available outcome measures received the treatment or control condition allocated) received 78% overall rater agreement between the authors [LTB and CKB], where the remaining questions received a 100% overall rater agreement between the authors [LTB and CKB].

**Table 4:** Quality Assessment of Included Studies (*N* = 9)

Quality item	Suo et al. [19] 2016	Rosen et al. [18] 2011	Lampit et al. [16] 2015	Belleville et al. [21] 2014	Lin et al. [17] 2014	Strenziok et al. [24] 2014	Lövden et al. [23] 2010	Antonenko et al. [20] 2016	Heinzel et al. [22] 2014
PEDro Scale Items									
1	+	+	+	+	+	−	+	+	−
2	+	+	+	+	+	+	−	−	−
3	+	+	+	+	+	−	−	−	−
4	−	+	+	+	+	+	+	−	−
5	+	+	+	−	−	−	−	−	−
6	+	−	−	−	−	−	−	−	−
7	+	+	+	−	+	−	−	−	−
8	+	+	−	+	−	+	+	+	+
9	+	+	−	+	−	+	+	+	+
10	+	+	+	−	−	+	+	−	−
11	+	+	+	+	+	+	+	+	+
Additional Items									
12	+	+	+	+	+	+	+	+	+
13	+	−	−	−	−	−	−	−	−
14	+	−	−	−	−	−	−	−	−

PEDro scoring system: receive a point (+) for each item that is met. When criteria were not met (−), no points were given

The maximum number of points is 10, which means excellent quality based on PEDro’s quality assessment

Additional Quality Assessment Items: Maximum score of 3

PEDro Scale

1. Eligibility criteria were specified (this item is not used to calculate the PEDro score)

2. Subjects were randomly allocated to groups

3. Allocation was concealed

4. The groups were similar at baseline regarding the most important prognostic indicators

5. There was blinding of all subjects

6. There was blinding of all therapists who administered the therapy

7. There was blinding of all assessors who measured at least one key outcome

8. Measures of at least one key outcome were obtained from more than 85% of the subjects initially allocated to groups

9. All subjects for whom outcome measures were available received the treatment or control condition as allocated or, where this was not the case, data for at least one key outcome was analyzed by “intention to treat”

10. The results of between-group statistical comparisons are reported for at least one key outcome

11. The study provides both point measures and measures of variability for at least one key outcome

Additional Items

12. Was cognition measured to assist the interpretation of neuroimaging results?

13. Was there a sample size calculation?

14. Was the compliance reported?

Of the three additional items, selected by the authors [LTB and TLA], item 12 (inclusion of cognitive outcomes to assist neuroimaging interpretation) was addressed by all nine studies [16–24]. Items 13 (sample size calculation) and 14 (reported compliance) were not addressed by eight studies [16–18, 20–24].

## Discussion

Findings from two high-quality studies examining the effect of CCT on *volumetric changes*, suggest that multi-domain CCT programs with a duration ranging from 12 to 26 weeks could result in an increase in grey matter density [16], but in contrast could also result in a decrease in cortical thickness in the posterior cingulate [19]. This indicates that in a relatively short time span, multi-domain CCT might be able to alter brain structure. However, the overall heterogeneity of the findings between studies (i.e., potential functional improvements versus declines), which could be in part due to the differences in region of interest, makes it difficult to draw definitive conclusions regarding the effect of CCT on brain structure.

*Task-based functional brain activity* decreased after training of a single task [21]; however, an increase in task-based brain activation was found in a more complex dual-task training [21] and a multi-domain CCT program [18]. This highlights that the CCT method (i.e., multi-domain versus single domain CCT) may play a critical role in task-based functional brain activity. Conversely, multi-domain CCT did not result in changes in *structural connectivity* [16], where an auditory perception-training program resulted in increased AD [24]. *Resting-state functional connectivity* was found to increase [16, 19] or decrease [16, 19, 24] depending on training type (e.g., single- versus multi-domain) and region of interest. Below, we will discuss as to why we might see a discrepancy between single- and multi-domain CCT effects, and why this discrepancy might affect both structural and resting-state functional connectivity differently.

### Task-based functional activity

Functional activation patterns in the brain change with aging as a result of neurophysiological changes. Compared with younger adults, functional activation patterns become less coordinated and localized in older adults, which result in loss of cognitive performance [25]. In the current review, three studies looked at functional activity in the brain while performing a task in the scanner. Activity levels in the brain while performing a task were both increased and decreased, depending on the type of training and region of interest. All three studies focused on different brain regions, which makes comparison difficult. However, results suggest that engaging in a more diverse or complex training (e.g., multi-domain CCT or dual-task training) might lead to an increased functional activation [18, 21] compared with training of a single task [21, 22]. In contrast, a short report focusing on transfer of training showed results that five weeks of training (i.e., letter memory and updating tasks) resulted in increases in task-related functional activity in the striatum compared with a passive control group [26]. Though, besides the focus on different brain regions, the vast differences in study design, such as the training duration, the presence or absence of a control group, and the small number of studies ask for prudence for making assumptions.

### Structural connectivity and type of training

DTI is an imaging technique used to determine the white matter microstructure of the brain by looking at how water molecules diffuse within the brain (i.e., the direction and amount of diffusion) [27, 28]. DTI is often quantified by measures such as FA and MD; which provide information about the direction of diffusivity and molecular diffusion rate, respectively. Decreases in FA and increases in MD might indicate lower levels of myelin or the presence of axonal injury, as water molecules are able to diffuse more freely (i.e., isotropic) [29, 30]. However, rather than looking at one specific DTI scalar (e.g., FA, MD), scalars need to be combined with other neuroimaging measures (e.g., T2, PD, FLAIR) to give a more detailed and accurate picture of for example white matter abnormalities that might occur within the brain [30]. Studies have linked loss of white matter integrity, as measured with DTI, to be associated with age-related cognitive decline in otherwise healthy older adults [31]. In addition, a meta-analysis focusing on DTI in MCI and Alzheimer’s Disease found increased MD in both MCI and Alzheimer’s Disease, as well as decreased FA in Alzheimers’ Disease compared with controls. More severe levels of Alzheimer’s Disease (i.e., lower scores on the Mini-Mental State Examination) were associated with reductions in FA [32].

Few studies looked at the effect of CCT on structural connectivity using DTI. One study of moderate-to-high quality (PEDro score of 7/10) found no changes in structural connectivity after 12 weeks of multi-domain CCT, which could be due to the small sample size [16]. These findings are in contrast with a quasi-experimental study [22] that found that an average of 100 h of training over four months resulted in decreased MD and increased FA in the genu of the corpus callosum. These findings suggest that multi-domain CCT is able to alter white matter microstructure in the brain in older adults. This finding could be promising as disruptions in white matter organization are often paired with cognitive decline [33]. However, a limitation of this quasi-experimental study is the lack of an active control group. Thus, we need more high quality studies to replicate these findings and to examine how multi-domain CCT might be able to alter white matter microstructure.

Increases in AD in the right occipito-temporal white matter were found in a study examining the effect of an adaptive auditory perception computer game (i.e., single-domain). This increased AD was correlated with a lower score in everyday problem solving and spatial working memory accuracy [24]. However, due to the absence of an included control group, this study used contrasts between the three training groups to look at improvements between groups. Therefore, results will more likely provide information about the effect of the training groups in relation to each other (i.e., which intervention shows the best results), than give information whether the intervention actually works.

### Functional connectivity and type of training

Resting-state fMRI is used to map networks in the brain, such as the well-established Default Mode Network (DMN) and the Central Executive Network (CEN). These networks are activated in both the presence [34] or the absence of a (cognitive) task [35, 36]. In patients with MCI or Alzheimer’s Disease, these functional networks in the brain are found to be disrupted [37, 38]. In addition, we can measure functional networks in the brain while performing a task with task-based fMRI.

Two studies [16, 19] showed that a multi-domain CCT intervention resulted in increased resting-state functional connectivity of the hippocampus. One high quality study (i.e., PEDro score of 9/10) found that a 26-week multi-domain CCT program alone (versus combination of CCT with resistance training) resulted in increased resting-state functional connectivity between the hippocampus and the left superior frontal lobe [19]. Additionally, a study with the same CCT program (i.e., COGPACK) found that multi-domain CCT resulted in increased resting-state functional connectivity between the right hippocampus and the left superior temporal gyrus after only three weeks of training [16]. These improvements in resting-state functional connectivity were significantly correlated with improved memory performance [19] and changes in global cognition at follow-up [16], respectively.

In accordance, an RCT of multi-domain CCT in older adults with a history of a stroke [17] found that CCT increased resting-state functional connectivity between the hippocampus and both the inferior frontal gyrus and the middle frontal gyrus. These increases in resting-sate functional connectivity were associated with significant positive changes in memory quotient and processing speed (Trail Making Test-A). Literature shows that resting-state functional connectivity between the hippocampus and the superior frontal lobe is reduced in MCI [37, 38]. Therefore, the current findings might indicate that multi-domain CCT could lead to improved cognitive performance through strengthening hippocampal functional networks and preventing memory loss that might be manifested by loss in hippocampal functional connectivity. However, the biological underpinnings of this change in connectivity are still unclear. Current histological findings suggest training induced neuroplasticity could be a result of dendritic branching, synaptogenesis or other factors such as angiogenesis [39]. Besides more human studies, we need to combine knowledge acquired from both human and animal (histological analyses), to help understand how multi-domain CCT could result in these functional changes in the brain.

Immediate comparison between the results of a single- versus multi-domain program can be made within one quasi-experimental study [24]. Participants in this study were randomly assigned to one of three included cognitive training programs. Participants who were randomized in Brain Fitness, a training program considered more single-domain in nature, showed decreased resting-state functional connectivity between the superior parietal cortex and the inferior temporal lobe. In contrast, participants who were assigned to Rise of Nation, a more multi-domain training, showed increased resting-state functional connectivity between the superior parietal cortex and the inferior temporal lobe. This contrast could be due to the nature of the training (i.e., single-domain versus multi-domain), as another quasi-experimental study [22] of single-domain CCT showed no changes in task-based functional connectivity following training.

A recent study [40] comparing non-computerized single-domain and multi-domain training found that multi-domain cognitive training mainly resulted in increased memory proficiency, while single-domain training primarily – but not only - enhances visuospatial and attentional benefits. Results of the current systematic review are in accordance with these findings, as the multi-domain CCT shows improvements in resting-state functional connectivity of hippocampus-frontal lobe and hippocampus-temporal lobe, which was associated with improvements in memory. Single-domain CCT did not result in similar findings. Gains in cognition resulting from multi-domain were more prone to sustain compared to gains acquired in single-domain cognitive training. Thus, multi-domain cognitive training might result in more widespread gains in cognitive functions, which maintain visible over a longer period of time compared to single-domain cognitive training.

### Quality assessment

The quality of studies was heterogeneous. Commonly missed criteria, were those that focused on blinding of participants, blinding of individuals who delivered the CCT, and blinding of the assessors. These issues could result into bias (i.e., either positively or negatively) during training and follow-up measurements due to expectations of both study examiners (treatment delivery or assessors) and participants. However, five [20–24] of the nine included studies were quasi-experimental and therefore the key characteristic of the more superior RCT, randomization into either an experimental or a control group, was lacking in these studies. The absence of a proper control group in these five quasi-experimental designs affects the interpretation of the results of the study; instead of whether a treatment works, quasi-experimental studies provide information on whether an intervention is more effective than a standard or alternative treatment.

Finally, of the three additionally included quality assessment criteria (i.e., item 12–14) two criteria (i.e., sample size calculation, compliance reported) were only met by one study [19]. The absence of sample size calculations and reported compliance in the remaining studies [16–18, 20–24], could result in a lack of power, which increases the chances of false negatives (i.e., type-II errors). This could mean that potential effects of CCT on neuroimaging parameters simply could not be detected due to a small sample size, and not because they were not present.

### Limitations

The studies included in this systematic review varied vastly in study design and CCT delivery, which resulted in a great deal of heterogeneity mainly in outcomes of functional and structural connectivity. Only four of the nine included studies were RCT’s [16–19]. However, the type of control group used varied; some studies included active controls, whether other control groups were of a passive nature (i.e., usual care). The inclusion of a control group, with a preference for the so-called active control groups, is recommendable in future studies. In addition, the heterogeneity of the findings in this systematic review might also be due to the large variability in type of training (single- versus multi-domain) and the dosage and duration of training (i.e., days versus months). Thus, the heterogeneous nature of the study designs in this review makes it difficult to draw conclusions. To better understand the relevant mechanisms of CCT, neuroimaging outcomes need to be accompanied with behavioural data. Furthermore, there are limited investigations regarding the transfer effects of CCT and the pattern of neuroplasticity associated with transfer. A high-quality study design, which includes for example an active control group, a literature-based training duration and dosage, and a sample size calculation, would help increase the consistency and comparability of findings, which in turn would help increase the ability to draw appropriate conclusions.

## Conclusions

This systematic review is an essential first step towards understanding the complex volumetric and functional changes, as well as changes in structural and functional connectivity that underlie CCT in older adults. However, the highly heterogeneous nature of the results in this systematic review, potentially due to the large variability in study design, indicates that more high-quality studies are needed to confirm and expand upon these findings. In addition, these studies do not provide information regarding the physiological and cellular mechanisms causing these structural changes. More histological studies are needed to gain insight whether these CCT induced changes might be a result of for example neurogenesis or synaptic plasticity. Future studies should focus on multi-domain CCT, since this type of training has the potential to induce more widespread and long-lasting effects on cognition.

## Abbreviations

AD: Axial diffusivity
BF: Brain fitness
BOLD: Blood oxygen level dependent
CCT: Computerized cognitive training
CEN: Central executive network
DMN: Default mode network
DTI: Diffusion tensor imaging
FA: Fractional anisotropy
fMRI: functional magnetic resonance imaging
ITL: Inferior temporal lobe
MCI: Mild cognitive impairment
MD: Mean diffusivity
MRI: Magnetic resonance imaging
PEDro: Physiotherapy evidence database
PRT: Progressive resistance training
RCT: Randomized controlled trial
RON: Rise of Nation
rsfMRI: resting-state functional magnetic resonance imaging
SF: Space fortress
SPL: Superior parietal cortex
